# A Systematic Review on Reporting of Methods in National Surveys about Adults’ Attitudes to Lifestyle and Environmental Risk Factors for Cancer

**DOI:** 10.3390/ijerph20095755

**Published:** 2023-05-08

**Authors:** Stéphane Faury, Philémon Aurouet, Bruno Quintard, Jérôme Foucaud

**Affiliations:** 1 Institut National du Cancer (INCa), Research in Social & Human Sciences, Public Health and Epidemiology Department, 92100 Boulogne-Billancourt, France; 2 Laboratory of Psychology EA 4139, 33405 Bordeaux, France; 3 Health Education and Practices Laboratory (LEPS EA 3412), Paris 13 University-UFR SMBH, 93017 Bobigny, France

**Keywords:** cancer, risk factor, attitude, systematic review

## Abstract

The risks of developing cancer are significantly affected by our lifestyle and environment. While there are several uncontrollable risk factors, we can modify our lifestyle and our environment to reduce the increased threat of cancer. This systematic review aims to evaluate the methodological assessment used to evaluate attitudes about cancer risk factors among the general population. Two researchers independently screened the articles for inclusion and Critical Appraisal Skills Programme (CASP) checklists were used to assess the methodology of the included studies. Thirty-one manuscripts met the inclusion criteria with a majority of them focusing on attitudes to several cancer risk factors and six on specific cancer location risk factors. This systematic review highlights the diversity of notions used around attitudes and methods used in the method of administering the survey, as well as the format of the questions and the response scales. It is thus difficult to compare data between different countries. However, cancer is a global problem. Harmonizing methods could allow a comparison of data between countries. Recommendations to this effect are suggested.

## 1. Introduction

Cancer is a significant global public health problem, with approximately 18 million new cases and more than 9.6 million deaths from the disease in 2018 [1]. The number of cancer cases has been increasing steadily over the last 10 years worldwide, with a 33% increase in the number of cases between 2005 and 2015 [2]. Research has shown that certain risk factors may increase a person’s chances of developing cancer [3]. Around the world, researchers have estimated the proportion of cancer cases attributable to lifestyle and environmental factors [4,5]. Between 30 and 50% of cancers may be preventable by modifying or avoiding key lifestyle and environmental risk factors [6]. The modifiable risk factors include: smoking (active and passive), alcohol, diet, overweight, obesity, infections, occupational exposures, ultraviolet radiation, ionizing radiation, physical activity, hormones, breastfeeding, outside air pollution, arsenic, and benzene [7].

The risks of developing cancer are significantly affected by lifestyle choices, which are particularly influenced by our social environment. There are several uncontrollable risk factors, but we can modify our lifestyle and act at our environmental level to reduce the increased threat of cancer [8]. Primary prevention and health promotion through lifestyle and environmental interventions remain the main way to reduce the global burden of cancer [9]. It is widely agreed that protective behavior can prevent many cancers, and knowledge is a necessary predisposing factor for such behavioral change [10]. In the public health research literature, notions of *knowledge*, *awareness*, *belief*, *representation*, *perception*, or *attitude* are sometimes juxtaposed. Although these words are frequently used interchangeably without posing a problem, they sometimes appear to have different intended meanings [11]. Whereas Trevethan (2017) recommended that “knowledge” be used to refer to information that is detailed and factual and that awareness be associated with personally relevant information, other authors [11,12,13] have used the term “attitude” which seems to include all of these concepts. For these reasons, in our article, we will systematically use the term *attitude.*

Several national surveys have been conducted to better understand attitudes about cancer risk factors, notably environmental factors (e.g., Health Information National Trends Survey, *Baromètre du Cancer*). For both decision-makers and researchers, it is important to know attitudes about cancer risk factors among the general population to guide public health policies and health communication strategies and to design lifestyle and environmental interventions. It is therefore necessary to identify national surveys reporting people’s attitudes about cancer risk factors, in particular, to point out the methodological similarities and divergences to provide an international working basis for future surveys about people’s attitudes about cancer risk factors, and to identify possible elements of comparability between studies and countries. To our knowledge, the reporting of methods used by national surveys to evaluate attitudes about cancer risk factors has not been systematically assessed.

To fill this gap, the French National Cancer Institute (INCa), which is the preeminent health and science agency in charge of cancer control in France, piloted a systematic review to evaluate the methodological assessment used to evaluate attitudes about cancer risk factors among the general public. Based on the highlighted results, recommendations to assess the perception of cancer risk factors can then be suggested.

## 2. Materials and Methods

According to French regulations, IRB approval is not required for this study.

### 2.1. Search Strategy

A search in the Prospero database (https://www.crd.york.ac.uk/PROSPERO/ (accessed on October 2020)) showed that no literature review is currently in progress on this subject (search terms: risk factors AND cancer AND knowledge OR awareness OR perception). This systematic review was guided by the Preferred Reporting Items for Systematic Reviews and Meta-Analysis (PRISMA) [14].

A search was performed from 1 May 2021 using the following databases: PubMed, PsychINFO, and PsycARTICLES, limiting the search to national surveys of adults (>18 years at diagnosis) published in English or French in peer-reviewed journals. Search terms are presented in Box 1 and the research equations used in PubMed (see Appendix A) were adapted for the other databases.

Box 1 **Search terms used in the databases.**

Representation* OR Awareness OR Perception* OR Knowledge* OR belief* OR attitude*

AND

risk factor or risk factors

AND

neoplas* OR cancer* OR tumor OR tumors OR tumour OR tumours OR malign* OR adenocarcinoma* OR carcinoma*

### 2.2. Inclusion and Exclusion Criteria

Qualitative, quantitative, or mixed design studies were included if they fulfilled the following criteria: (a) assessed environmental and lifestyle risk factors for cancer among the general public; (b) conducted on people aged 18 and over; (c) written in English or French; (d) published in peer-reviewed journals.

Predefined exclusion criteria were: (a) reviews, case–control studies, protocol or pilot studies and studies that evaluated cancer risk factors and not cancer risk factor awareness or the awareness of risk factors for specific cancers (e.g., breast cancer); (b) unpublished papers due to the lack of peer-review oversight; (c) studies on cancer patients or their families, or people at risk of developing cancer; (d) protocol studies or studies that described and/or evaluated the effectiveness of an intervention on knowledge and lifestyle for cancer prevention; (e) cohorts that were not representative of a defined population; (f) studies focusing on a subgroup of the main sample.

### 2.3. Study Selection and Data Extraction

Duplicate articles were removed. Title and abstract screening was conducted according to the eligibility criteria. We obtained full articles for all titles that appeared to meet the inclusion criteria. Full-text reviewing was conducted by two different researchers (Stephane Faury and Jerome Foucaud) to establish a final list of eligible studies. Data extraction was performed by S.F. and checked by J.F. In cases of disagreement, a third researcher (Bruno Quintard) was requested for an opinion, and a consensus was reached among the researchers (Stéphane Faury, Jérôme Foucaud, and Bruno Quintard). Additional papers were searched using the reference list of eligible studies. Data collection was performed with the aim of including: authors, year, country, sample, study design and methods, objective(s), main results, and limitation of the method. Extraction included all reporting items used in the studies. Items other than the main preventable cancer risk factors were classified into several categories by Philémon Aurouet, and a consensus was reached among the other authors on this categorization.

### 2.4. Critical Appraisal of Study Quality

The methodological quality of included studies was independently assessed by two researchers (S.F. and J.F) using the Critical Appraisal Skills Programme (CASP) checklists [15,16,17]. In cases of discrepancies, the manuscripts were discussed verbally. CASP checklists consist of three sections (Section A: “Are the results of the trial valid?”; Section C: “What are the results?”; Section C: “Will the results help locally?”). As the CASP checklist does not provide a total score for each study, based on Lamore et al. (2019) [18], we chose to classify the studies as either (1) a low-quality study (i.e., participants not recruited in an acceptable way and weak results), (2) a medium-quality study (i.e., participants recruited in an acceptable way and weak to moderate results) or (3) a high-quality study (i.e., participants recruited in an acceptable way and strong results).

## 3. Results

**Figure 1:**
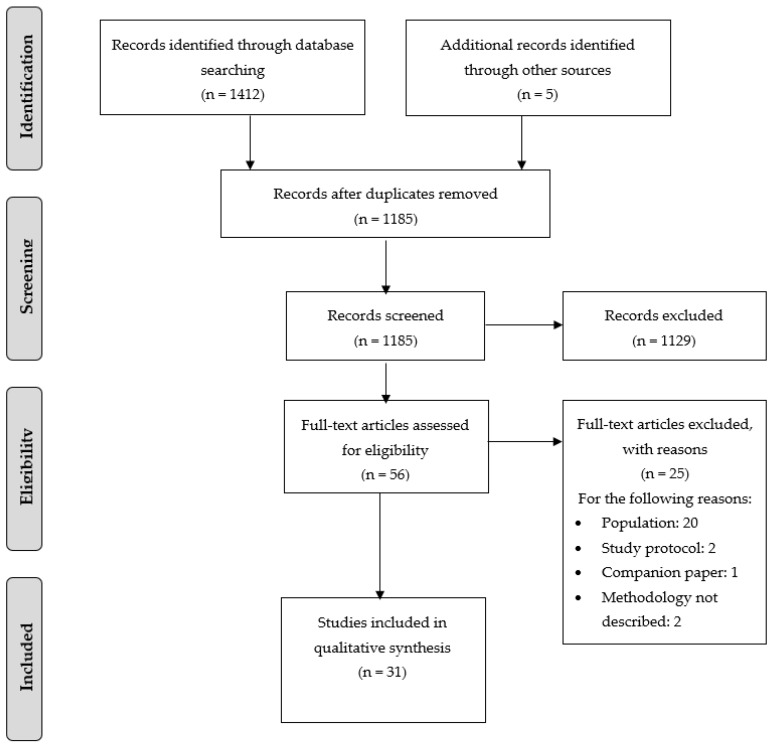
Flow diagram of study selection according to PRISMA.

The initial search yielded 1417 records. After the removal of duplicates, the titles and abstracts of 1185 records were screened; 1129 records were identified as clearly non-relevant and were thus excluded: 56 were retained for full-text analysis. Finally, 31 articles [19,20,21,22,23,24,25,26,27,28,29,30,31,32,33,34,35,36,37,38,39,40,41,42,43,44,45,46,47,48,49] were included in this systematic review without any disagreement (i.e., inter-rater agreement = 100%). For two articles [22,43], we contacted the study authors to obtain additional information but did not receive any responses. Figure 1 presents a flow diagram of the research article selection process.

### 3.1. Study Design and Participant Characteristics

The 31 cross-sectional studies included in the review were published between 1992 and 2021 and conducted around the world (see Table 1). The majority of the studies focused on attitudes to cancer risk factors (*n* = 24 out of 31; [20,21,23,24,26,27,28,29,30,31,33,34,35,36,37,38,39,40,41,43,44,45,46,47,48]) and six others focused on attitudes to risk factors for many specific cancers (for example, for breast, cervical, prostate and colon cancer) [19,22,25,32,42,49]. In total, 112,904 participants (from 358 to 19,076 participants), mostly women, were included in these studies. A description of the study included in this review is presented in Appendix B.

### 3.2. Quality Assessment of the Included Studies

The results of the quality assessment are summarized in Table 2. Very high inter-rater agreement was obtained. We are unable to answer questions Q6a and Q6b as to whether the follow-up of subjects was complete enough.

*Four cohort studies were classified in the “low-quality”* [25,40,43,47] *group because the participants might not have been recruited in an acceptable way and this may compromise the extent to which the findings can be generalized (see answers to Q2). The aims of the Ryan et al. study* [43] *and the Shi et al. study* [47] *were to assess public perception of the risk factors for cancer. However, participants were recruited through social media platforms, which represents a major limitation for the representativeness of the general population. For the Raj et al. study* [40]*, clear information on participant recruitment is not presented. The Daley* [25] *study was designed to ascertain college students’ knowledge about risk factors. Recruitment was performed in one university: The author distributed surveys to undergraduate students at a large public university in the Northeastern United States. Only students present on the day that the surveys were handed out were surveyed; as such, absentees were not given another chance to fill them out. Seventeen studies were classified into the “medium-quality” group* [19,22,23,24,26,27,31,32,35,36,39,41,42,45,46,48,49] *because (1) exposure was not accurately measured to minimize bias (see answers to Q3); (2) the pilot test was not used to establish the reliability and validity of the questionnaire for new questions (see answers to Q4); (3) the most important confounding factors were not identified and/or taken into account in the design and/or analysis (see answers to Q5a and Q5b); (4) the studies had no precise results (see answers to Q8); (5) the design and methods of the study were sufficiently flawed to make the results unreliable (see answers to Q9); (6) the results cannot be applied to the local population (see answers to Q10). Finally, ten cohort studies were classified into the “strong-quality” group* [20,21,28,29,30,33,34,37,38,44].

### 3.3. Objective(s) of Study about Cancer Risk Factors?

In the public health research literature, the terms “knowledge”, “awareness”, “belief”, “representation”, “public perception”, or “attitude” are sometimes juxtaposed. In this systematic review, twelve articles focused on “awareness” [20,26,28,31,33,40,41,43,44,46,47,49], eight on “knowledge” [21,22,25,27,30,36,37,39], six on “perception” [19,23,34,35,38,42], one on “knowledge and belief” [24], one on “awareness and belief” [29], two on “knowledge and attitude” [32,45] and one on “perception and belief” [48] (see Appendix B).

The majority of studies focused on several cancer risk factors [20,22,25,26,27,28,30,31,32,33,35,36,37,38,39,40,41,42,43,44,45,46,47,48,49]. Six studies focused on specific cancer risk factors: dietary and environmental factors [19,34], overweight and obesity-related cancer risk factors [23,34], alcohol factors [21,24] and age factors [29].

### 3.4. Instrument Administration

Different methods were used to administer questionnaires: interview (*n* = 12) [20,22,27,28,31,32,36,39,40,41,44,49]; computer-assisted telephone interview (CATI) (n = 9) [29,30,33,34,35,38,42,46,48]; online survey (*n* = 3) [21,43,47]; self-reported (*n* = 2) [37,45]; telephone surveys [23,24]; sent questionnaire (*n* = 1) [19]; distributed questionnaire for one survey [25] (description in Appendix B). The data are missing for one study [26].

### 3.5. How Are Attitudes to Risk Factors Assessed?

Some studies used open-ended questions [27,30,39,42,42,44]. Close-ended questions were used in many studies but differed according to their pre-defined responses. Some articles used a list of cancer risk factors [19,22,25,41,43,49]. Others used Likert scale multiple-choice responses about the importance of risk factors (for example, “probably not very important” to “very important” or “very likely” to “very unlikely”) [19,23,34,41]; the extent to which factors increase or decrease cancer risk [19,35]; and the degree of agreement [24,29,33,38,43,46,48]. Some studies proposed dichotomous questions (yes/no; true/false; risk factor/preventive factor) [20,21,26,32,36,43]. For one study, the responses were the attributable proportion of cancer causes [31]. For one dichotomous question (yes/no), participants who answered “yes” were then asked an open-ended question [23]. For five studies, data about how attitudes to risk factors are assessed are missing [28,37,39,40,45]. Three studies used both open-ended and closed-ended questions [23,27,39]. A complete description of studies’ attitudes’ assessment methods is presented in Appendix B. Four studies used validated standardized measures: the Awareness and Beliefs about Cancer (ABC) questionnaire [33,46,47] and the Cancer Awareness Measure (CAM) questionnaire [27].

The ABC questionnaire is a reliable, validated instrument for measuring knowledge and beliefs about cancer (see Table 3) [50]. The core measure includes 32 ‘core’ items (open-ended and closed-ended questions): (1) awareness of cancer symptoms, (2) awareness of cancer outcomes, (3) help-seeking intentions, (4) beliefs about cancer, (5) beliefs about barriers to symptomatic presentation and (6) estimated age at which people are most likely to develop cancer. The optional modules are modules on cancer screening and awareness of risk factors for cancer [50]. The 13 risk factors for cancer are: smoking, exposure to another person’s smoke, drinking more than one unit of alcohol a day, eating less than five portions of fruit and vegetables a day, eating red/processed meat, obesity, sunburn in childhood, being over 70 years old, having a close relative with cancer, infection with human papillomavirus (HPV), low physical activity, using sunbeds, and exposure to ionizing radiation.

The CAM questionnaire is a validated standardized measurement for cancer awareness in the general population (see Table 3) [51]. The CAM consists of 47 items (open-ended and closed-ended questions): (1) warning signs; (2) seeking medical advice; (3) barriers to seeking medical advice; (4) risk factors; (5) cancer and age; (6) most common cancer; (7) awareness of NHS screening programs. One open-ended question has been designed to enquire about risk factors: “What things do you think affect a person’s chance of getting cancer?” and 11 closed-ended questions have been designed to measure a respondent’s level of agreement with the 11 risk factors: “These are some of the things that can increase a person’s chance of developing cancer. How much do you agree that each of these can increase a person’s chance of developing cancer?”. The 11 risk factors are: smoking any cigarettes at all, exposure to another person’s cigarette smoke, drinking more than one unit of alcohol a day, eating less than five portions of fruit and vegetables a day, eating red or processed meat once a day or more, being overweight (BMI over 25), getting sunburnt more than once as a child, having a close relative with cancer, being over 70 years old, infection with HPV, and performing less than 30 min of moderate physical activity five times a week.

The number of cancer risk factor reporting items used in the studies varied greatly, ranging from 1 to 128 items used [19,21,29]. Questions focused on: cancer-reducing strategies [23,24,30,36,41,42] and/or things that cause a person to develop cancer or increase their chances of developing cancer [19,20,20,22,23,24,25,26,27,28,29,31,32,33,34,35,37,38,39,40,41,42,43,44,45,46,47,48,49].

Most of these studies used items related to the main preventable cancer risk factors [1]: smoking [19,20,22,23,25,26,27,28,31,32,33,35,36,37,38,39,40,41,43,45,46,47,49], alcohol [19,20,21,24,25,26,28,31,32,33,35,36,37,38,39,40,41,43,45,46,47], diet [19,20,22,23,25,26,27,28,31,32,33,34,35,36,37,39,40,41,43,45,46,47,49], overweight and obesity [19,20,23,25,26,27,28,31,32,33,34,35,40,41,43,46,47,49], infections [19,25,28,32,33,37,40,41,46,47,49], occupational exposure [25,31], ultraviolet radiation [19,20,23,27,27,28,32,33,35,38,41,45,46], ionizing radiation [19,28,32,33,37,46], insufficient physical activity [19,23,26,27,31,32,33,34,35,36,38,41,43,46], hormones [19,25,28,41,49], breastfeeding [26,34,43], outside air pollution [19,31,35,38,45], and arsenic or benzene [32].

A large number of items were used to assess cancer risk attitudes among the general population with regard to other environmental and endogenous factors, along with specific health aspects. Most of these items were related to probable risk factors or assessed common beliefs about non-presumptive factors. Other environmental factors that were assessed were related to: diet, such as specific foods (33 items: such as fatty foods [23,26,35,45,49], salt [26,34,35,43], charred meat or fish [31,35], green tea [26,43], vitamins and minerals [35,43]) and processed food ingredients (additives and preservatives [19,28,31,35,49], artificial sweeteners [19,32], food coloring [26]); exposure to toxins, such as chemicals (14 different items such as: antiperspirant [25,32,41], chemical substances in general [19,25,28], pesticides [19,24,31], aerosols [19,43], and hair dyes [19,32]), pollution (industrial [38,40], environmental [39], and general pollution [49]), and radiation [28]; water treatments [19]; electromagnetic fields (high-voltage power lines [19,32,35,41,49], mobile phones [32,35], mobile phone relay stations [38], and microwave ovens [19]); other waves (loud music [35], computer screen [19], TV screen [19], fluorescent light [19]); medical procedures (breast implant [25], Pap smear [25], tooth filling [19], and vasectomy [25]) and medication (aspirin [19], cough medicine [19], sleeping pills [19]); animal-related causes [19]; and finally, bacteria and parasites [19,28,31].

Endogenous factors were assessed through three main item categories: age, expressed as aging [20,22,32,33,41,49], being 35 or 50 years old [45], and being 70 years old [27,28,29,46,47]; heredity, expressed as having close relatives with cancer [27,33,41,45,46,49], a family history of cancer [19,20,22,32,40,47]; and genetics [25,31,43].

Specific health aspect factors were also assessed and grouped into four categories: sexual and reproductive health/behaviors, such as having many/multiple sexual partners [19,22,24,25,40,41,49], having sex at a young age [25,40], early puberty [40], not using condoms [25] and poor sexual/genital health [40]; mental health, consisting of stress [19,28,31,35,38,41,49], good sleep hygiene [19,36], resentment caused by a personal or professional disappointment [38], general worries [19] or painful experiences [38]; women’s health, regarding late menopause [25,40], nulliparity [40], pregnancy [25], abortion [25], having a first child after 30 [25]: and finally, general lifestyle and behavioral responsibility [19,48].

Finally, 13 items could not be classified, such as: wearing underwire bras [25,43], wart or mole irritation [45], poverty [25] or luck [43].

## 4. Discussion

This systematic review aims to evaluate the methodological assessment used to evaluate attitudes about cancer risk factors among the general public.

The results of this systematic review show that different methods were used. The main method used to administer questionnaires is the interview. The advantage of interview-administered surveys is that respondents had the opportunity to seek clarification if they did not understand a question [28]. Online surveys [21,43,47] are also used but the findings may not be representative of people who do not access the Internet [21]. However, Connor et al.’s [52] study compares risk factors between data collected online and face-to-face. Comparisons of data collected using face-to-face interviews and online surveys revealed minor differences between samples [52].

In 2007, the NHS Cancer Reform Strategy emphasized the importance of raising awareness of early warning signs and risk factors of cancer among the general population. The CAM tool [51] was developed to help measure levels of cancer awareness, explore risk factors for poor cancer awareness, and develop and evaluate interventions to promote cancer awareness [53]. CAM data were first collected in 2008 and subsequently every two years up to 2014. In 2014, the CAM tool was modified to include additional questions. A trend analysis was conducted comparing data from 2008–2014. In 2017, data were collected online and face-to-face. Results were compared to gain an understanding of differences according to the data collection method. Based on these results, the CAM survey was moved online in 2019. Only the 2008 version of CAM has been validated. In this systematic review, of the 20 studies published in 2010 or after, only two [27,47] used the CAM questionnaire and only one used it to evaluate the attitude about cancer risk factors [27]. Elshami’s study used 8 items of the 11 items proposed by the CAM questionnaire. Items about drinking more than a unit of alcohol a day, eating red or processed meat once a day or more, and HPV infections are excluded.

Internationally, there are variations in cultural attitudes to cancer (e.g., public education about cancer, delivery of healthcare, etc.), and these may shape attitudes about cancer. Simon et al. [50] only found one validated measure of cancer awareness: CAM, which does not include items on beliefs or attitudes and has not been assessed internationally. An internationally valid measure of attitude about cancer is essential to take this research forward [50]. The ABC questionnaire was designed for this purpose. In this systematic review, three studies used the ABC questionnaire [29,33,46,47]. Item selection for the ABC tool was informed by theoretical frameworks outlining processes of patient delay, the English Department of Health’s National Awareness and Early Diagnosis pathway and existing surveys such as the CAM questionnaire [50]. The optional module on awareness of risk factors for cancer included 13 items: 11 from the CAM questionnaire and two further items (using a sunbed and exposure to ionizing radiation).

In this systematic review, of the 15 studies published in 2013 or after, only four [29,33,46,47] used the ABC questionnaire and only three used it to evaluate attitudes toward cancer risk factors. Only one used the 13 items of the original version [33]. Shi et al. [47] used 10 of the 13 items; the physical activity risk factor was omitted from their final questionnaire due to an error when transcribing the survey into the web survey tool. The items regarding using a sunbed and exposure to ionizing radiation were excluded. Thus, except for the physical activity items, the items used in Shi et al. [47] to measure attitudes about cancer risk factors are the 11 common items between the CAM questionnaire and the ABC questionnaire. Forbes et al. [29] used the ABC questionnaire to assess the recognition of cancer symptoms.

The ABC tool was designed to be administered by telephone interview in order to be practical for data collection across diverse geographic areas [33]. The CAM tool was designed to be administered as an interview, either face-to-face or over the telephone. The CAM tool may be used on the Internet, or as a ‘self-reported’ survey without supervision (e.g., by mail) but these options will provide lower-quality data [53]. Thus, the CAM questionnaire can be used in many study designs, whereas the ABC questionnaire seems to be used only for telephone interviews.

The ABC tool is a reliable and valid measure of cancer awareness and beliefs. Validated versions have been developed for six countries (UK, Australia, Canada, Sweden, Denmark and Norway) and in five languages (English, Canadian French, Swedish, Danish and Norwegian). The CAM questionnaire was developed for the UK population, although some authors (e.g., Elshami et al. [27]) translated the CAM questionnaire from English to another language.

Given that most focused on general cancer risk factors, a wide variety of items were observed among the studies. Assessing environmental/behavioral and endogenous cancer risk factors, these studies mainly addressed attitudes among the general population towards preventable risk factors. Smoking, alcohol, diet, and overweight/obesity were assessed by around two-thirds of the included studies. However, on account of the diversity of methodologies used, and the heterogeneity of these studies, it is not possible to compare their results. Although it is necessary to understand attitudes among the general population to the main cancer risk factors, some studies did not include all main risk factors: For instance, Elshami et al. [27] used the CAM survey but removed the item on alcohol even though it is the second most important preventable risk factor. In the studies retrieved in this review, there appears to be a lack of homogeneity in the items used to compare the assessment of attitudes on the main preventable cancer risk factors. Nevertheless, Shi et al. [54] used a 10-item version of the ABC questionnaire that ultimately resembles the CAM questionnaire. Furthermore, while they are not responsible for the most attributable fraction, some of the main preventable cancer risk factors were not sufficiently included in the surveys in the studies, such as infections, occupational exposures, and ionizing radiation. This list of established cancer risk factors is based on the current state of epidemiological knowledge and may be subject to change as exposure evaluation methods develop. Environmental synergies such as “cocktail effects” need to be better documented and cancer awareness reporting surveys should take these environmental questions into consideration. Some studies proposed unestablished and suspected environmental cancer risk factors. There is a need for better homogeneity and priority setting in terms of including suspected cancer environmental risk factors when measuring attitudes among the general population.

Few studies used open-ended questions. Closed-ended questions have a limited set of possible answers and questions are often good for surveys, because higher response rates are obtained. Prompted questions receive significantly higher recognition than open-ended questions that rely on recall [35], but closed-ended questionnaires prevent respondents from qualifying or justifying their responses [38]. Moreover, the closed-ended question format is also challenging for researchers because it may be assumed that they know more about a survey topic than they do. Closed-ended questions come in a multitude of forms. In this systematic review, we observed different formats such as the Likert scale multiple-choice question responses about the importance of risk factors (for example, “probably not very important” to “very important” or ‘to very likely’ to “very unlikely”); the extent to which factor increase or decrease cancer risk; and the degree of agreement. Some studies proposed dichotomous questions: Yes/No; True/False; Risk factor/Preventive factor. There are almost as many studies as there are response methods, which makes it difficult to compare the results because the information levels are not the same.

This systematic review highlights the diversity of methods used both in the method of administering the survey as well as the format of the questions and the response scales. It is thus difficult to compare data between different countries. However, cancer is a global problem. Harmonizing methods could make it possible to compare data between countries. This could also make it possible to build prevention campaigns based on international data. In accordance with the findings of this systematic review, we present a schematic representation of the steps researchers should follow to build a study to evaluate attitudes about cancer risk factors among the general public, along with recommendations:

(1) Regarding the terms used, in studies included in this systematic review, to evaluate attitudes about cancer risk factors among the general public. Some studies used awareness (*n* = 12), others knowledge (*n* = 8) or perceptions (*n* = 6). Authors do not define the term used, so we do not know whether different terms are used to refer to the same concept. We recommend that authors define the concept used and what they want to evaluate. If the term is defined, it will be possible to make a comparison between studies.
(2) Regarding “How questions about risk factors are determined”, the authors used previously standardized and validated tools (e.g., ABC, CAM), or the questions were based on prior cancer research, international literature and other cancer-related population surveys. We also recommend presenting the work teams in the article, including, in addition to researchers and health experts, representatives of patient organizations. To build a questionnaire, it is also important to know the concerns of the target population concerning risk factors and not just researchers’ concerns.
(3) Regarding question format: We suggest that for some questions for which the findings need clarification, closed-ended and open-ended formats can be asked for the same question. For example, in the Cancer Barometer 2015, almost 10% of respondents were of the view that some cancers are contagious, but the closed-ended question used does not make it possible to elaborate on this result. In 2020, the same closed-ended question was asked, but for participants who responded “Agree”, an open-ended question was asked to find out why they think that some cancers are contagious. In this systematic review, Cameron et al. [23] used one dichotomous question (Yes/No), but participants who responded “Yes” were then asked an open-ended question [23]. Moreover, a combination of open-ended and closed-ended questions is often used to compare spontaneous responses and respondents’ choices when lists of responses are provided. For example, in Cancer Barometer 2020, firstly, an open-ended question is asked: “What do you think the three main causes of cancer are?”, followed by a closed-ended question with 17 risk factors (Likert scale for each item: Certainly, probably, probably not, certainly not, don’t know).
(4) In this systematic review, few studies mentioned whether pilot testing was used or not. We recommend that pilot testing be used to establish the reliability and validity of the questionnaire for new questions.
(5) Regarding the method used to administer the questionnaire: It is important that the method include all participants so that the sample is representative of the general population (e.g., people who do not access the Internet; illiterate people, etc.). For Connor et al. [52], there are minor differences between data collected using face-to-face interviews and online surveys [52]; combining methods is possible.

This review has certain limitations. Firstly, even though our search was extensive, we cannot be certain that all relevant articles were included. Secondly, studies published in sources other than peer-reviewed journals were not included. We can assume that some authors have presented their work in non-scientific journals. Thirdly, we only included studies with a representative cohort of a defined population. Indeed, some design studies are excluded such as qualitative studies. Fourthly, studies focused on specific cancer risk factors were not included. Fifthly, our reporting item review might not be exhaustive as studies may have included items not mentioned in the article in their questionnaire items.

## 5. Conclusions

This systematic review highlights the diversity of methods used: method of administering the survey, question format, and response scales. It is thus difficult to compare data between different countries. However, cancer is a global problem. Harmonizing methods could allow a comparison of data between countries. Moreover, to assess trends in attitudes among the general population as well as the effectiveness of communication campaigns around the prevention of cancer risk factors, longitudinal studies should be considered.

## Figures and Tables

**Table 1:** Countries of included studies.

Country of Publication ^1^	Number	References
Australia	6	[19,21,23,24,29,35]
United-Kingdom	4	[29,41,44,49]
United States	4	[22,25,30,48]
India	3	[36,39,40]
Canada	2	[29,47]
Danish	2	[29,33]
France	2	[34,38]
Swedish	2	[29,33]
Turkey	2	[32,45]
Ethiopia	1	[20]
Iran	1	[28]
Ireland	1	[43]
Japan	1	[31]
Mexico	1	[37]
Morocco	1	[26]
New-Zealand	1	[42]
>>Palestine/Gaza	1	[27]

^1^ Some studies included multiple countries.

**Table 2:** CASP checklist results for assessing the methodological quality of the included studies.

Cohort Studies	Q1	Q2	?	Q3	Q4	Q5a	Q5b	Q6a	Q6b	Q7	Q8	Q9	Q10	Q11	Q12
Baghurst et al. (1992) [19]	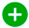	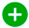	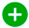	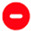	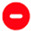	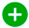	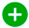	NA	NA	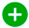	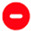	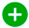	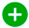	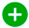	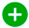
Bantie et al. (2021) [20]	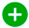	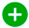	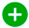	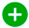	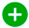	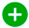	+/−	NA	NA	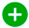	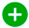	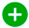	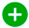	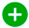	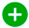
Buykx et al. (2015) [21]	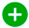	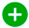	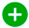	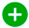	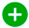	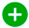	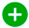	NA	NA	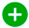	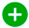	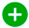	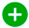	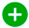	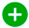
Breslow et al. (1997) [22]	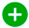	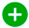	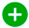	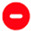	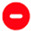	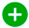	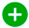	NA	NA	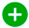	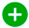	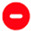	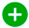	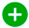	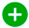
Cameron et al. (2010) [23]	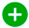	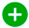	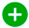	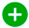	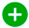	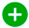	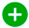	NA	NA	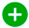	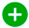	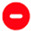	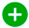	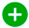	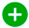
Cotter et al. (2013) [24]	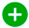	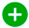	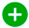	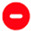	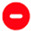	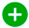	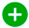	NA	NA	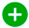	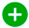	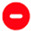	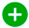	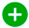	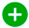
Daley (2007) [25]	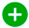	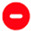	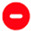												
El Rhazi et al. (2014) [26]	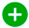	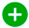	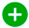	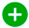	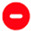	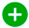	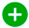	NA	NA	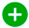	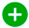	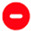	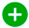	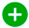	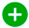
Elshami et al. (2020) [27]	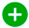	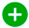	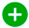	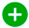	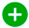	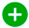	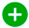	NA	NA	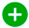	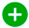	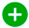	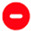	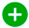	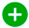
Feizi et al. (2010) [28]	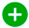	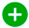	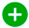	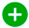	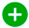	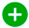	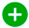	NA	NA	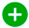	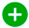	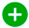	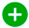	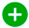	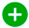
Forbes et al. (2013) [29]	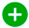	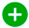	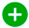	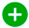	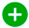	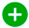	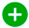	NA	NA	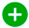	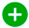	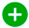	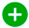	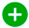	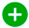
Hawkins et al. (2010) [30]	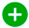	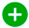	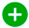	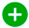	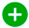	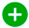	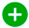	NA	NA	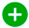	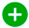	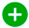	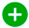	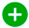	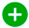
Inoue et al. (2006) [31]	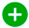	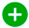	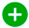	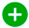	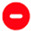	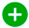	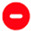	NA	NA	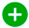	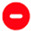	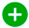	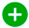	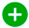	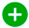
Karadeniz et Çetinkaya (2021) [32]	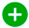	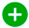	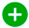	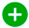	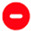	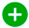	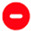	NA	NA	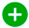	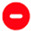	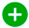	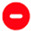	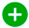	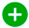
Lagerlund et al. (2015) [33]	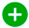	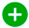	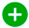	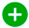	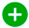	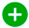	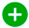	NA	NA	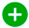	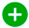	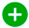	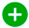	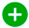	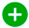
Lamore et al. (2019) [34]	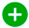	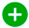	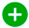	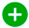	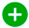	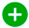	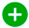	NA	NA	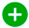	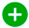	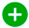	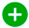	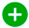	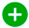
MacTiernan et al. (2014) [35]	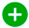	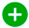	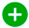	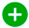	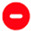	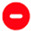	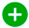	NA	NA	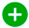	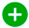	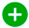	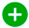	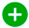	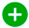
Oswal et al. [36]	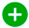	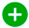	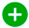	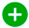	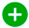	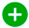	+/−	NA	NA	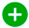	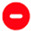	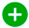	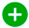	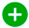	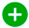
Perez-Contretras et al. (2004) [37]	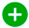	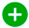	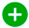	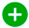	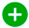	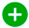	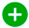	NA	NA	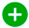	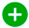	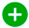	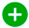	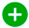	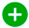
Peretti-Watel et al. [38]	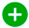	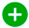	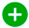	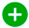	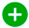	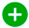	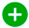	NA	NA	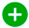	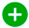	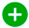	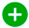	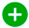	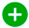
Puri et al. (2009) [39]	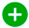	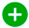	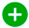	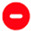	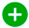	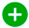	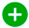	NA	NA	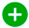	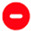	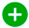	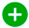	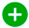	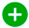
Raj et al. (2012) [40]	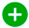	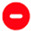	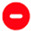												
Redeker et al. (2009) [41]	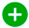	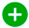	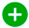	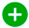	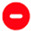	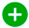	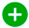	NA	NA	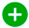	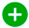	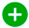	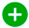	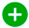	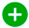
Reeder et al. (2003) [42]	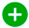	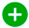	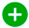	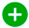	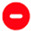	+/−	+/−	NA	NA	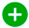	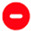	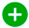	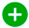	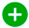	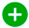
Ryan et al. (2015) [43]	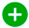	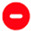	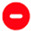												
Sanderson et al. (2009) [44]	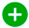	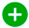	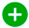	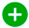	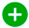	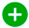	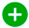	NA	NA	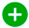	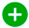	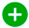	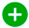	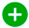	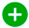
San Turgay et al. (2005) [45]	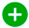	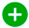	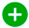	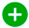	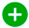	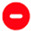	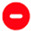	NA	NA	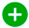	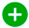	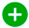	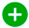	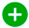	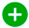
Schliemann et al. (2020) [46]	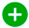	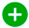	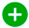	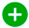	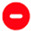	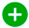	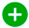	NA	NA	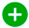	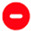	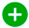	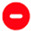	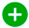	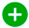
Shi et al. (2020) [47]	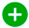	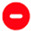	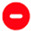												
Vanderpool et al. (2010) [48]	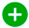	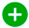	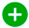	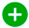	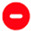	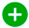	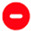	NA	NA	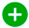	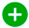	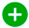	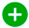	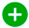	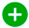
Wardle et al. (2001) [49]	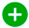	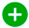	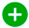	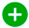	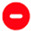	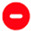	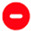	NA	NA	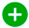	* 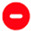 *	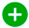	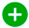	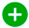	* 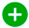 *

Question for cohort study assessment: Q1 = “Did the study address a clearly focused issue?”; Q2 = “Was the cohort recruited in an acceptable way?”; ? = “Is it worth continuing?”; Q3 = “Was the exposure accurately measured to minimize bias”; Q4 = “Was the outcome accurately measured to minimize bias?”; Q5a = “Have the authors identified all important confounding factors ?”; Q5b = “Have they taken account of the confounding factors in the design and/or analysis?”; Q6(a) = “Was the follow-up of subjects complete enough?”; Q6(b) = “Was the follow-up of subjects long enough?”; Q7 = “What are the results of this study?”; Q8 = “How precise are the results?”; Q9 = “Do you believe the results?”; Q10 = “Can the results be applied to the local population?”; Q11 = “Do the results of this study fit with other available evidence?”; Q12= “What are the implications of this study for practice?”. Answer key: 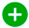; = yes or strong; 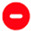; = no or weak; +/− = uninterpretable; NA = not applicable.

**Table 3:** Items in CAM and ABC questionnaires.

Topic	CAM	ABC
Smoking	Smoking any cigarettes at all Exposure to another person’s cigarette smoke	Smoking? Exposure to another person’s smoke?
Alcohol	Drinking more than one unit of alcohol a day	Drinking more than one unit of alcohol a day. One unit of alcohol is equivalent to a single measure of spirits, a third of a pint of normal-strength lager or beer, or a small glass of wine.
Diet	Eating less than five portions of fruit and vegetables a day Eating red or processed meat once a day or more	Eating less than five portions of fruit and vegetables a day. Eating red or processed meat once a day or more. By processed meat, I mean meat that is smoked, salted or chemically preserved.
Obesity/overweight	Being overweight (BMI over 25)	Being obese.
UV	Getting sunburnt more than once as a child	Getting sunburnt more than once as a child.
		Using a sunbed.
Age	Being over 70 years old	Being over 70 years old.
?	Having a close relative with cancer	Having a close relative with cancer.
Infections	Infection with HPV (Human Papillomavirus)	Infection with HPV, Human Papillomavirus.
Physical activity	Doing less than 30 min of moderate physical activity five times a week	Low physical activity.
Ionizing radiation		Exposure to radiation such as radioactive materials, X-rays or radon.

## Data Availability

Research data of this article are available in the manuscript.

## Appendix A. Research Equations Used in PubMed Database

((representation* [Title] OR awareness [Title] OR perception [Title] OR perceptions [Title] OR knowledge [Title] OR knowledges [Title] OR belief* [Title] OR beliefs [Title] OR attitude [Title] OR attitudes [Title]) AND (risk [Title] OR risks [Title] OR factor [Title] OR factors [Title]) AND (neoplas* [Title] OR cancer*[Title] OR tumor [Title] OR tumors[Title] OR tumour[Title] OR tumours [Title] OR malign*[Title] OR adenocarcinoma*[Title] OR carcinoma*[Title]) AND (“humans”[MeSH Terms] AND (English[lang] OR French[lang]) AND “adult”[MeSH Terms]).

## Appendix B. Description of the Methodology Used in the Included Studies

**Author (Year) [Ref.], Country**	**Socio-Demographic Data (n, n Male, Age, Education)**	**Study Design**	**Objective(s) of Study about Cancer Risk Factors?**	**How Are Attitudes to Risk Factors Assessed?**	**How Are Questions about Risk Factors Determined?**	**Limits of Methodology**
Baghurst et al. (1992) [19], Australia	n = 1500; n_male_ = NA Mean age: NA Level of education: NA	A questionnaire was sent with a self-addressed stamped envelope for questionnaire return, a cover letter explaining the purposes of the study, and a toll-free number for enquiries.	- Article focused on public perceptions. - Importance of specific risk factors for three major cancer sites (colon, lung, breast); - Perception of the importance of a wide range of potential environmental carcinogens, particularly those related to diet.	- Participants were asked to list, in order of importance, up to three risk factors from a list of nine for each cancer. Respondents were also allowed to enter additional risk factors if they wished. - Two sets of questions were asked to assess the respondents’ concerns about a wide range of potential environmental carcinogens: the first listing contained some 63 general items (household items, environmental pollutants, pills, medications, infections and lifestyle factors). Respondents were asked to score each item as “probably not very important”, “quite important”, “very important”, or “I’m not sure” concerning their role in the initiation or promotion of cancer. Non-preventable risk factors were also included in this list for comparison. The second listing concentrated entirely on individual foods and nutrients. Respondents were asked to indicate whether they felt that each of the 38 food/drink items or 21 nutrients “may increase”, “may decrease”, or “probably have no effect” on cancer risk or whether they had “no idea”.	NA	-
Bantie et al. (2021) [20] Ethiopia	n = 845; n_male_ = 313 Mean age: 29.8 years (SD, 10.4) Level of education: unable to read and write (11.5%), able to read and write (8.1%), primary school (15.3%), secondary school (32.9%) or college and above (32.2%)	Interview-administered surveys	- Article focused on awareness regarding risk factors. - Awareness of the person’s chance of developing cancer among Bahir Dar city residents.	A standardized and validated data collection tool for awareness regarding risk factors of cancer (9 items). For example, “smoking cigarettes can increase a person’s chance of developing cancer” (yes/no/don’t know).	NA	-
Buykx et al. (2015) [21] Australia	n = 2482; n_male_ = 1221 Mean age: 46.8 years (SD, 16.3) Level of education: year 10 or less (14.3%), year 11 or 12 (16.5%), diploma or certificate (36.7%), university degree (32.5%)	Online surveys	- Article focused on knowledge of cancer risk. Public support for a range of alcohol policies; associations between personal factors, including knowledge of alcohol as a cancer risk, and support for alcohol policies.	“Which of the following do you think can result from drinking too much alcohol?” Cancer—yes/no/don’t know).	The alcohol-specific section was compiled by the authors, utilizing previously validated tools where possible. The survey was pilot-tested for comprehensibility.	Findings may not be representative of people who do not access the Internet and/or opt to join a panel.
Breslow et al. (1997) [22] US	n = 12,035; n_male_ = NA Mean age: NA Level of education: NA	Interview	- Article focused on knowledge of cancer risk. Americans’ knowledge of risk factors for breast, cervical, colon and prostate cancer.	Knowledge about risk factors was elicited by an interviewer who asked women “Which of these things do you think increases a woman’s chances of getting cancer of the breast?” Similar questions were asked for cancers of the cervix and colon. Men were asked similar questions for cancers of the colon and prostate. As the question was asked, respondents looked at a flashcard with seven possible answers: “1. Increasing age”, “2. High-fat diet”, “3. Low-fiber diet”, “4. Smoking”, “5. Family history”, “6. Having multiple sex partners”, and “6. None of these”. A response of “don’t know” was acceptable but not listed on the flashcard.	Knowledge about risk factors for cancer was assessed using the General Knowledge and Attitude section of the National Health Interview Survey (NHIS) Cancer Control Supplement. These questions were reviewed by cognitive scientists.	Respondents were prompted to recognize risk factors from a written list. It is likely that the percentage of subjects who properly identified risk factors would have been even lower had they been asked to recall the risk factors without the benefit of a predefined list.
Cameron et al. (2010) [23] Australia	n = 1433; n_male_ = 513 Mean age: NA Level of education: year 9 or below (14.4%), year 10 or 11 (22.40%), year 12 (31.70%), tertiary degree (31.5%)	Telephone surveys	- Article focused on perceived risk factors for cancer. Adults’ knowledge and beliefs regarding behavioral risk factors for cancer.	Respondents were asked, “Do you believe there are any things people can do to reduce their risk of cancer?” (Yes/No). Those who answered “yes” were then asked, “What would be one thing that people could do to reduce their risk of cancer?” and then “What other things can be done?” If food or diet was mentioned, respondents were prompted to indicate, “What things should people eat more of to reduce their risk of cancer?” and “What things should people eat less of to reduce their risk of cancer?” Respondents were allowed to name up to three things people should eat more and less of, respectively. In addition, respondents were specifically asked about the importance of various factors in increasing a person’s risk of cancer. Responses were recorded on a four-point scale ranging from “very important” to “not at all important”, with respondents also given the option “don’t know/can’t say”.	Questions were based on prior CBRC (Centre for Behavioural Research in Cancer) research and other cancer-related population surveys conducted.	NA
Cotter et al. (2013) [24] Australia	n = 1255; n_male_ = 604 Mean age: NA Level of education: <High school (21.9%), high school (42.3%), tertiary (35.8%)	Telephone surveys	- Article focused on knowledge and beliefs. Knowledge and beliefs about longer-term health risks related to alcohol consumption among Australian adults.	All participants were asked whether they thought cancer (and other diseases) result from drinking too much alcohol. They were also asked whether they agreed that “Limiting your alcohol intake helps prevent cancer” (agree strongly/somewhat vs other).	NA	NA
Daley (2007) [25] US	n = 3362; n_male_ = 1143 Mean age: 20.25 years (SD, 2.62) Level of education: NA	Surveys were distributed to students	- Article focused on knowledge of risk. College students’ knowledge of risk of breast, cervical, and testicular cancers.	A list of risk factors was created. Author coded answers into correct versus not correct.	Based on preliminary research conducted during the 2001–2002 academic year (interviews and a free list of risk factors)	Only students present on the day the surveys were handed out were surveyed; absentees were not given another chance to fill them out.
El Rhazi et al. (2014) [26] Morocco	n = 2891; n_male_ = 1433 Mean age: 41.60 years (SD, 15.20) Level of education: illiterate (43.5%), <6 years school (29.1%), > or =6 years school (27.4%)	NA	- Article focused on public awareness. Cancer risk factors knowledge of the Moroccan population	The people’s knowledge of cancer risk factors was assessed by choosing the correct answer from three responses (risk factor/protective factor/don’t know) for each of the 14 proposed factors.	The questionnaire contained questions on the awareness of various cancer risk factors according to international literature.	The questionnaire did not give people the option of saying that a factor is neither risky nor protective.
Elshami et al. (2020) [27] Palestine	n = 1429; n_male_ = 705 Mean age: 33.7 years (SD, 11.4) Level of education: NA	Face-to-face interview.	- Article focused on knowledge level of cancer risk factors in the Gaza strip.	Arabic version of the Cancer Awareness Measure (CAM) (8 items of the 11 items of the CAM awareness of risk factors for cancer module) A 5-point Likert scale was used to assess the awareness of cancer risk factors. The questionnaire comprised open-ended (recall) questions and closed-end questions (recognition).	CAM is a validated instrument	NA
Feizi et al. (2010) [28] Iran	n = 2500; n_male_ = 1175 Mean age: NA Level of education: lack of education (2.1%), less than diploma (24.8%), diploma (42.3%), university (30.8%)	Interview-administered surveys	- Article focused on public awareness. Iranian awareness of risk factors for cancer.	Questionnaire consisting mainly of closed-ended questions about the awareness of various environmental and lifestyle risk factors for cancer (n = 12). Each answer related to knowledge of cancer is assigned a score of 0 or 1.	The questionnaire was pilot-tested to establish the reliability and validity of the questionnaire and to verify data collection methods, particularly to determine the appropriateness of its format, level of difficulty, and length of time to complete the questionnaire	The survey was interview-administered so respondents had the opportunity to seek clarification if they did not understand the question.
Forbes et al. (2013) [29] Australia, Canada, Denmark, Norway, Sweden, UK	n = 19 079; n_male_ = 7775 Mean age: NA Level of education: lack of education (2.1%), less than diploma (24.8%), diploma (42.3%), university (30.8%)	Computer-assisted live telephone interviews	- Article focused on awareness and beliefs. Cancer awareness and beliefs between six countries.	“Cancer risk is higher in people aged ≥70 years than at a younger age”	NA	NA
Hawkins et al. (2010) [30] US	n = 5589; n_male_ = NA Mean age: NA Level of education: <high school (16.2%), HS graduate 30.2%, some college (26.2%), college graduate (23.5%), DK/ref/miss (3.9%)	Computer-assisted telephone interview (CATI) system is administered in English or Spanish.	- Article focused on public knowledge. Cancer-related knowledge, beliefs, attitudes, and behaviors among the adult population in the United States.	Open-ended questions: “Can you think of anything people can do to reduce their chances of getting cancer?” “If participants cited one cancer-reducing strategy, they were then asked, “Anything else” (maximum of 9 answers). Respondents’ open-ended responses were coded to fit into one of the 17 response categories.	Questions were developed specifically for HINTS and were based on the HINTS conceptual framework. Response categories were defined at the time of survey development and tested for validity through a field test.	Respondents had a limited amount of time to think about and cite strategies for reducing cancer risk and no cues to help them recall specific prevention strategies.
Inoue et al. (2006) [31] Japan	n = 1355; n_male_ = 609 Mean age: NA Level of education: junior high school (14.9%), senior high school (51.2%), college or higher (33.9%)	Face-to-face interview	- Article focused on public awareness. Awareness of the attributable fraction of cancer causes among the Japanese.	The first question asked about the preventable fraction of cancer which would result in Japan if each factor were completely and totally eliminated, using the fine categories of <5%, 5 to <10%, 10 to <15%, 15 to <20%, 20 to <25%, 25 to <30%, 30 to <40%, 40 to <50%, 50 to <60%, 60 to <70%, 70 to <80%, 80 to <90%, and 90 to 100%. These categories were shown together on a pie chart. The second question asked about the fraction of cancer genetically predetermined using the same categories as the first. The third asked about the preventable fraction of cancer by modification of lifestyle using estimation of an actual per cent value.	These risk factor candidates were selected from previous international and domestic recommendations and guidelines	This appears to be the first attempt to discover the level of awareness for each risk factor candidate, and the questionnaire used has hence not been fully validated.
Karadeniz et Çetinkaya (2021) [32] Turkey	n = 1200; n_male_ = 600 Mean age: 54.9 years (SD, 11.2) Level of education: primary school and lower education level (54.9%), NA	Interview with a questionnaire at home	- Article focused on knowledge levels and attitudes about cancer risk factors and the risk factors for the cancer types (breast, cervical, ovarian, stomach, colon, and skin).	Items with response options of yes/no or no idea.	The researcher developed a questionnaire after reviewing the literature and receiving expert opinions.	NA
Lagerlund et al. (2015) [33] Sweden and Denmark	Denmark: n = 3000; n_male_ = 1341 Mean age: 55.9 years (SD, 13.3) Level of education: primary and lower secondary (18.9%), upper secondary (46.9%), Bachelor and PhD (34.2%), missing (<1%) Sweden: n = 3070; n_male_ = 1352 Mean age: 56.6 years (SD, 14.1) Level of education: primary and lower secondary (18.4%), upper secondary (40.8%), Bachelor and PhD (40.8%), missing (<1%)	Computer-assisted telephone interview (CATI)	- Article focused on awareness. Awareness of several established risk factors for cancer between a Danish and a Swedish population.	Awareness and Beliefs about Cancer (ABC) measure I am now going to read out a list of things that may or may not increase your chances of getting cancer. For each one can you tell me how much you agree or disagree that it may increase your chances of getting cancer? The response options were dichotomized into awareness (tend to agree and strongly agree) and lack of awareness (tend to disagree, strongly disagree, and don’t know)	ABC is a validated instrument	NA
Lamore et al. (2019) [34] France	In 2010: n = 3345; n_male_ = 1465 Mean age: NA Level of education: <high school (46.4%), high school (19.7%), university level (33.9%) In 2015: n = 3345; n_male_ = 1718 Mean age: NA Level of education: <high school (53%), high school (19.1%), university level (27.9%)	Computer-assisted telephone interview (CATI)	- Article focused on the perception of factors associated with cancer risk in the French population in 2010 and 2015. Regarding the links between diet, physical activity, obesity and breastfeeding factors and cancer risk.	Two main multiple-choice questions were presented: (1) Do you think that diet has a “very important”, “somewhat important”, “somewhat unimportant” or “not at all important’ role in cancer development? (2) In your opinion, frequent consumption of “e.g., red meat, milk, etc.” “can lower”, “can increase”, or “has no influence on’ cancer risk.	NA	NA
MacTiernan et al. (2014) [35] Australia	n = 2094; n_male_ = 872 Mean age: NA Level of education: NA	Computer-assisted telephone interview (CATI)	- Article focused on public perceptions. Perceptions of cancer risk factors among Western Australian adults.	Seventeen diet-related risk factors: Of these, eight factors were established cancer risk factors, five factors were unestablished or mythical and four were in a ‘contestable’ category: ‘Do you think eating any of the following types of food on a regular basis increases, decreases or has no effect on cancer risk?’ Sixteen environmental or lifestyle-related risk factors: Of these, seven factors were established cancer risk factors, seven factors were unestablished or mythical and two were in a ‘contestable’ category: ‘Which of the following do you think increase, decrease or have no effect on cancer risk?’ The response categories were ‘increase a little’, ‘increase a lot’, ‘decrease a little’, or ‘decrease a lot’.	The category for each factor was based on the Cancer Council Western Australia website (the primary source of information on cancer risk factors for Western Australian adults)	Prompted questions receive significantly higher recognition than open-ended questions that rely on recall
Oswal et al. (2020) [36] India	n = 1400; n_male_ = 705 Mean age: NA Level of education: No education (8%), up to primary (30%), up to secondary (51%), more than secondary (11%)	Interview with questionnaire	- Article focused on public knowledge about factors that reduce cancer risk.	Six questions about risk factors associated with cancer (yes, no, don’t know).	The questionnaire was adapted from the previously published literature in the Indian context on assessing the knowledge level of cancer among the population in community-based and hospital-based settings. The questionnaire was pilot-tested.	The limitation is the questionnaire design (yes, no or don’t know). Participants with low education and low confidence may be more likely to say they know nothing, fearing that if they say they know something, they might be questioned further and might say incorrect things.
Perez-Contretras et al. (2004) [37] Mexico	n = 13,293; n_male_ = 5848 Mean age: NA Level of education: NA	Self-reported questionnaire	- Article focused on knowledge about cancer risk factors. Knowledge about cancer risk factors among public school students in Mexico.	10 questions about risk factors associated with cancer	Questions were based on previous studies carried out on Mexican and other populations.	Because the data were self-reported by the students, the results could underestimate levels of knowledge about cancer risk factors.
Peretti-Watel et al. (2019) [38] France	n = 3359; n_male_ = 1472 Mean age: NA Level of education: without diploma (8.69%), < high school (37.57%), high school (19.68%), > high school (34.06%)	Computer-assisted telephone interview (CATI)	- Article focused on perceptions of cancer risk factors.	14 items on perceptions of risk factors for cancer. For each item, respondents were asked to report whether they thought the factor could increase a person’s risk of developing cancer (certainly not, probably not, probably, certainly, don’t know/no response). General opinion on cancer: “Nothing can be done to avoid cancer” (strongly disagree, somewhat disagree, somewhat agree, strongly agree, don’t know).	The questionnaire was pilot-tested.	Closed-ended questionnaire prevents respondents from qualifying or justifying their responses. People living in retirement homes, hospitals or other institutions were excluded from the survey.
Puri et al. (2009) [39] India	n = 1350; n_male_ = NA Mean age: NA Level of education: Illiterate (18.3%), primary (5.5%), matric (26.8%), 10 + 2 (17.5%), graduate (18.1%), post-graduate (14.6%)	Interview Questionnaire was completed by a team of medical social workers, interns, and doctors	- Article focused on knowledge of cancer. Knowledge, attitude, and practices about various aspects of cancer.	Open and closed-ended questions NA	The investigating tool used was a preformed, pretested questionnair.	NA
Raj et al. (2012) [40] India	n = 3070; n_male_ = NA Mean age: NA Level of education: NA	Interview NA	- Article focused on awareness. Awareness regarding risk factors.	NA *	NA	NA
Redeker et al. (2009) [41] UK	n = 4233; n_male_ = 1887 Mean age: NA Level of education: NA	Home interview	- Article focused on public awareness. Awareness of cancer risk factors.	Awareness of cancer risk factors was assessed by presenting respondents with a list (including well-established risk factors as well as some with no established link to cancer) and asking, “Which of these things do you think increase a person’s chance of developing some types of cancer”. Respondents could choose as many items as they wished Beliefs about the impact of lifestyle changes on the risk of cancer were assessed with the question “How likely do you think it is that a person can reduce their chances of getting cancer sometime in their life by making changes to their lifestyle?” Response categories were “very likely”, “quite likely”, “neither likely nor unlikely”, “quite unlikely”, “very unlikely”, and “don’t know”. Beliefs about personal cancer risk-reducing behavior were assessed by asking respondents to name steps they could take to reduce their cancer risk.	List created by CRUK	
Reeder et al. (2003) [42] New Zealand	n = 438; n_male_ = 207 Mean age: NA Level of education: NA	Computer-assisted telephone interview (CATI)	- Article focused on perceptions. Adults’ perceptions of the causes and primary prevention of common fatal cancers (breast, cervical, prostate, melanoma, bowel and lung cancer).	Presentation of questions was dependent on sex. For each cancer: “Do you know of anything that increases the risk of getting “e.g., breast” cancer?” For breast/prostate cancer: “In what age group do you think a [woman/man] is most likely to develop breast/prostate cancer?” For melanoma: “Have you or anyone else deliberately checked your skin for changes which could be melanoma or other skin cancer in the last 12 months?” For lung cancer: “How much do you think that a regular smoker can reduce their risk of lung cancer by quitting smoking?”	Questionnaire content drew from multiple sources.	
Ryan et al. (2015) [43] Ireland	n = 748; n_male_ = 100 Mean age: 37 years (SD, 19) Level of education: Completed secondary school education (92%), undergraduate degree (31%), postgraduate degree (21%)	Online (SurveyMonkey)	- Article focused on awareness. Awareness of risk factors for cancer.	Forty-eight question survey about 12 specific diet-related and 14 lifestyle-related risk factors “True or false: cancer risk increases with age”. Open-ended question: “In your opinion, what are the top 5 risk factors for cancer?”, a total of 12 different answers were given. “Diet has a significant role to play in cancer prevention.” Participants were given a list of potential behaviors and asked to either agree or disagree that they were risk factors for cancer.	Questions were constructed based on a thorough literature review and using the eight recommendations published in the WCRF report	
Sanderson et al. (2009) [44] UK	n = 1747; n_male_ = 826 Mean age: NA Level of education: None (30.6%), GCEs (32.85%), A-levels (22.7%), degree (13.85%)	Interview NA	- Article focused on awareness. - Awareness of lifestyle risk factors for cancer	Open-ended question about cancer risk factors: “What do you think are the things that cause a person to develop cancer or increase their chances of developing it” Respondents were encouraged to list as many risk factors as they could. Responses were coded according to 1 of 26 pre-defined categories.	Item was adapted from previous research (Waller et al., 2004)	Occupational environments were not coded for in these analyses
San Turgay et al. (2005) [45] Turkey	n = 358; n_male_ = 209 Mean age: 40.27 years (SD, 7.99) Level of education: NA	Self-reported questionnaire	- Article focused on knowledge and attitudes. Knowledge of cancer among schoolteachers working in Turkey.	Closed-ended question Each response is assigned a score of 0 to 1. The questionnaire includes a total of 15 questions but only one about cancer risk factors: What is the first factor that increases catching cancer?	Authors developed the instrument and pretested it on a group of schoolteachers. It had not been used previously	Data were self-reported with no objective measures available to evaluate teachers
Schliemann et al. (2020) [46] Malaysia	n = 1895; n_male_ = 1082 Mean age: NA Level of education: No formal education (1.5%), primary education (8.6%), secondary education (55.5%), tertiary education (34.5%)	Computer-assisted telephone interview (CATI)	- Article focused on risk factor awareness in Malaysia.	Awareness and Beliefs about Cancer (ABC) measure (12 items of the 13 items of the ABC awareness of risk factors for cancer module) “‘I am now going to read out a list of things which may or may not increase your chances of getting cancer in general. For each one can you tell me how much you agree or disagree that it may increase your chances of getting cancer?’ with the possible answers ranging from strongly agree to strongly disagree on a 5-point Likert scale.	ABC is a validated instrument.	NA
Shi et al. (2020) [47] Canada	n = 1019; n_male_ = 237 Mean age: NA Level of education: Did not complete high school (3.7%), high school (18.8%), college diploma or university degree (59.1%), graduate, post-graduate, or professional degree (18.4%)	Web-based survey using a self-reported questionnaire.	- Article focused on cancer risk factor awareness.	The Awareness and Beliefs about Cancer (ABC) instrument (10 items of the 13 items of the ABC awareness of risk factors for cancer module) and one more item added by the research team.	ABC is a validated instrument	The ABC instrument had one risk factor awareness question on physical activity that was omitted from the final questionnaire due to an error when transcribing the survey into the web survey tool. As an online survey was used, we were not able to assess the response rate and make necessary comparisons between respondents and non-respondents. Even then, those who are more aware of cancer risks or are more concerned about their own risk of cancer may have been more likely to respond to the survey compared to those who have low awareness or concern. Some participants’ information was self-reported, and thus the possibility of bias in the accuracy of recall information could not be ruled out.
Vanderpool et al. (2010) [48] US	n = 7674; n_male_ = 629 Mean age: NA Level of education: NA	List-assisted random digit dial CATI and mail survey	- Article focused on risk perceptions and beliefs. Cancer risk perceptions in Appalachia.	It seems like almost everything causes cancer. Agree/Disagree Cancer is most often caused by a person’s behavior/lifestyle. Agree/Disagree.	NA	
Wardle et al. (2001) [49] UK	n = 3693; n_male_ = 1600 Mean age: NA Level of education: NA	Interview	- Article focused on awareness. Awareness of risk factors for breast, cervical, prostate, bowel, and lung cancer.	Respondents were asked to identify risk factors for breast, cervical, prostate, bowel, and lung cancer from a list of 14 which included both established causes and so-called mythic causes.	NA	
* In this article, the Materials and Methods section is not detailed.						
